# Six-month effects of the Caregiver Support Model on reducing family caregivers’ needs and enhancing their resources

**DOI:** 10.1093/geroni/igaf122.4192

**Published:** 2025-12-31

**Authors:** Ka Hung Edwin Chung, Kin-Kit Li, Dannii Yeung

**Affiliations:** City University of Hong Kong, Hong Kong, China (People’s Republic); City University of Hong Kong, Hong Kong, China (People’s Republic); City University of Hong Kong, Hong Kong, China (People’s Republic)

## Abstract

Caring for frail older adults imposes significant stress and burden on family caregivers. To address these challenges and enhance their resources, the Caregiver Support Model (CSM) was developed (Leung et al., under review). The model incorporates systematic assessment, personalized intervention plans, and sustained support for caregivers. This longitudinal study examined the long-term effects of the CSM on 162 family caregivers from nine elderly centers (Mage=69.4, SD = 11.8; 79.6% female) between July 2024 and July 2025. Participants were initially assessed for their caregiving needs and resources using the Caregiver Needs and Resources Assessment (CNRA) (Li et al., 2023), followed by the implementation of personalized intervention plans over six months. Data were collected at baseline, three months, and six months. Controlling for demographic factors of both caregivers and care recipients, repeated measures MANCOVA revealed that the intervention program significantly reduced caregiver needs, including physical needs, role conflict, care recipient needs, psychological, and social support needs [F(10, 880) = 6.00, p <.001]. Additionally, caregiver resources — include spirituality, self-efficacy, responsibility, community resources, family support, understanding of care recipients, and a healthy lifestyle — showed significant improvement over the three timepoints [F(14, 876) = 6.85, p <.001]. Improvements were more pronounced at six-month compared to three-month, indicating a lasting effect and consolidation of gains. These findings underscore the importance of personalized interventions that take caregivers’ evolving needs into consideration through systematic assessment. The CSM presents a promising approach to enhancing caregiver well-being and managing the complex demands of caregiving, particularly in an aging population.

